# Investigation of I*b*-Values for Determining Fracture Modes in Fiber-Reinforced Composite Materials by Acoustic Emission

**DOI:** 10.3390/ma14133641

**Published:** 2021-06-29

**Authors:** Doyun Jung, Woong-Ryeol Yu, Wonjin Na

**Affiliations:** 1 Composite Materials Applications Research Center, Korea Institute of Science and Technology (KIST), 92 Chudong-ro, Bongdong-eup, Wanju-gun, Jeonbuk 55324, Korea; jungdoyun@kist.re.kr; 2 Department of Materials Science and Engineering (MSE), Research Institute of Advanced Materials (RIAM), Seoul National University, 1 Gwanak-ro, Gwanak-gu, Seoul 08826, Korea; woongryu@snu.ac.kr

**Keywords:** fiber-reinforced composites, acoustic emission, fracture, I*b*-values

## Abstract

This study analyzed failure behavior using I*b*-values obtained from acoustic emission (AE) signals. Carbon fiber/epoxy specimens were fabricated and tested under tensile loads, during which AE signals were collected. The dominant peak frequency exhibited a specific range according to fracture mode, depending on the fiber structures. Cross-ply specimens, with all fracture modes, were used and analyzed using *b-* and I*b*-values. The *b*-values decreased over the specimens’ entire lifetime. In contrast, the I*b*-values decreased to 60% of the lifetime, and then increased because of the different fracture behaviors of matrix cracking and fiber fracture, demonstrating the usefulness of I*b*-values over *b*-values. Finally, it was confirmed that abnormal conditions could be analyzed more quickly using failure modes classified by I*b*-values, rather than using full AE data.

## 1. Introduction

Fiber-reinforced plastics (FRPs) are widely used in various fields of engineering—such as aerospace, ships, and machinery—because of their high specific strengths [1,2,3]. The global composite materials market is expected to grow to USD 113.6 billion by 2024 [4]. The mechanical properties of FRPs are determined by the fiber structure, exhibiting anisotropic mechanical behavior. Therefore, it is essential to consider and analyze failure modes (e.g., matrix cracking, interfacial failure, and fiber fracture), considering the fiber structure in the design of FRP materials [5,6,7,8]. A proper non-destructive test is required in order to monitor the damage that occurs during FRP testing and relate it to failure modes [9,10,11,12,13].

Acoustic emission (AE) testing [14,15] is a suitable method for monitoring the fracture phenomena of a material. AE is a transient elastic wave emitted from the crack, which is recorded as an electrical voltage (waveform) through a piezoelectric sensor attached to the surface of the material. AE testing uses only the elastic wave generated from the crack, without the injection of external energy, to evaluate the condition of the crack in real time. In addition, continuous monitoring of complex structures is possible through AE testing [16]. Researchers have utilized the fast Fourier transform and wavelet transform methods to investigate AE signals for damage mode analysis [17,18]. Compared with the conventional fast Fourier transform method, the wavelet transform method is more useful in that it can simultaneously obtain frequency and time information. Suzuki et al. [19] used the wavelet transform method to classify AEs from unidirectional glass-fiber-reinforced plastic into four distinct failure modes. In carbon-fiber-reinforced plastic (CFRP), however, failure modes are not easily classified [20,21,22,23,24]; therefore, specific defects are first introduced using a designed CFRP (e.g., mainly matrix cracking: [90]n; almost no fiber breakage: [80]n; and all types of fracture: [0]n).

In AE testing, the cumulative hit trend and specific indices provide information helpful for evaluating the damage state during each moment. The *b*-value is a classic index of structural health related to crack size and growth; it is defined as the slope of cumulative hit distribution according to amplitude (Gutenberg–Richter formula), and can be used to evaluate both microcracks and macrocracks [25]. Moreover, the I*b*-value (suggested by Shiotani [26]) provides better reliability through statistical analysis of the amplitude distribution. Colombo et al. [27] analyzed the *b*-value of a reinforced concrete beam under cyclic loading, and revealed the relationship between *b*-value and damage state (lower *b*-value at macrocrack). The same study also suggested a *b*-value standard for the reinforced concrete beam: macrocrack opening (*b* > 1.7), macrocrack holding (1.2 < *b* < 1.7), and macrocrack propagation (1.0 < *b* < 1.2).

The *b*-value analysis tracks the amplitude trend of AE signals, showing a lower value at a severe damage state. To the authors’ knowledge, the application of *b*-value analysis in composite materials is rare, and is a first in our research group [28,29,30,31]. In previous studies in our group, we revealed a specific frequency AE signal range for each fracture mode, and the *b*-value analysis is feasible in composite materials. At the same time, we also found that the *b*-value can be affected by the propagation distance, or environmental factors such as UV irradiation, and the crucial factor is the high attenuation rate of composite materials. However, the detailed *b*-value analysis for each fracture mode was none, detecting the activation time and linkage between fracture modes. This analysis can provide helpful information about fracture behavior and the analysis approach, promoting our research.

**Figure 1:**
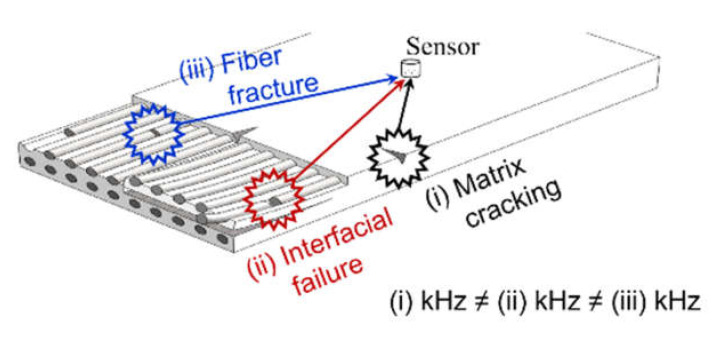
Schematic diagram.

In this study, the damage state and failure mechanism of CFRP were investigated under tensile loading. The technical issues, and the accompanying methodology, are schematically shown in Figure 1. CFRP specimens were prepared with a circular notch to specify the location of the crack. In the tensile test, the measured AE signal was converted via wavelet transformation to distribute the damage modes (i.e., Gabor wavelet, peak frequency) [18,32]. Finally, the fracture mechanism was analyzed according to the frequency-dependent damage modes, and I*b*-values were evaluated. This research provides a basis for correlations between AE and the fracture behavior of CFRP; it also establishes a technique for analyzing fracture behavior through AE testing.

## 2. Experimental

### 2.1. Materials and Specimen Preparation

It has been well established that the dominant fracture modes in FRPs during the tensile test are affected by their stacking sequences [20,21,22]. Table 1 shows the dominant fracture modes for tensile specimens with the stacking sequences [20]. Note that if the fibers are arranged parallel to the loading direction, it is the (0-degree) layer. We prepared CFRP specimens following these stacking sequences. First, we fabricated CFRP plates with prepreg (250 mm × 150 mm, Mitsubishi Rayon Co., Ltd., PYROFIL TR380G250S, Tokyo, Japan), performing a pre-curing process at 85 °C for 2 h and then curing at 135 °C for 3 h under a pressure of 0.7 MPa. Rectangular specimens were processed from these plates, and center holes were introduced (see Table 2 for specifications, ASTM D3039). The widths of specimens (0)_8_ and (0/90)_4_ were smaller than the widths of specimens (90)_16_ and (80)_16_, considering the differences in failure stress.

**Figure 2:**
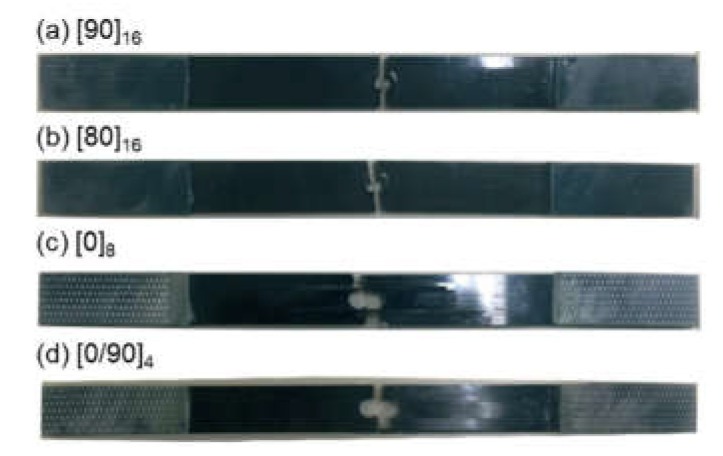
Carbon-fiber-reinforced plastic specimen after tensile testing.

*b*- and I*b*-value analyses should be conducted using AE signals originating from a specific location. When damage occurs at various locations in a structure, the damage severity differs with respect to the damage at the AE source location. In this study, a center hole was introduced to concentrate the locations of the AE sources. As shown in Figure 2, the specimens broke at or near the center hole, and no damage occurred in other areas of the specimens. Figure 2 shows that the failure mechanisms also changed with the stacking sequence, as demonstrated in Table 1.

### 2.2. Acoustic Emission Testing

**Figure 3:**
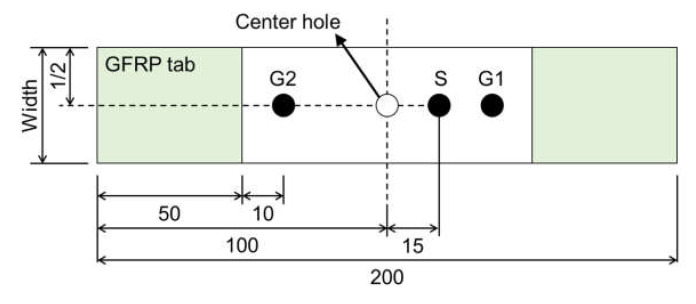
Schematic illustrations of fiber-reinforced plastic specimens with center hole and sensor locations (units in mm).

Before each tensile test, 2.5-mm-thick glass-fiber-reinforced polymer tabs were attached to both ends of each specimen in order to avoid any damage from the jig. Three AE sensors (Type PICO; Physical Acoustics, Princeton Junction, NJ, USA) were mounted on each specimen using silicone grease and vinyl tape. Figure 3 shows the sensor locations, labeled as “S”. The primary sensor was mounted 20 mm from the center of the hole. Two guard sensors were also attached at a distance of 10 mm from each tab (G1 and G2, see Figure 3). The sensor was used to investigate the influences of various FRP fracture modes on the *b*- and I*b*-values; the guard sensors were used to distinguish AE from outside noise. The cross-head speed of the tensile tester was controlled at 0.1 mm/min. AE signals larger than 40 dB_AE_ were detected using a digitizer (Physical Acoustics; Type PCI-2) at a sampling rate of 10 MHz per channel during each test (threshold: 40 dB_AE_, amplifier: 40 dB_AE_, pre-trigger: 50 μsec). AE testing conditions of the woven GFRP are given in [33].

### 2.3. Analysis of b- and Ib-Values

**Figure 4:**
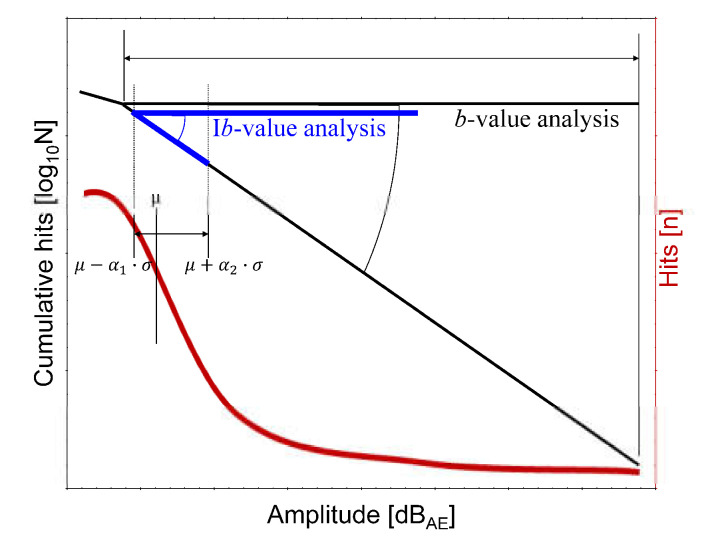
*b*- and I*b*-value analysis [28].

Gutenberg initially developed *b*-value analysis for seismological applications using the Gutenberg–Richter formula [25]:

(1) $$\log_{10}N=a-bM$$

where *N* is the total number of earthquakes stronger than *M* in a particular region over a defined period, a is an empirical constant, and *b* is the slope of the linear relationship. This *b*-value analysis was eventually extended to AEs from a material that fractures over a specific time interval under a defined load:

(2) $$\log_{10}N=a-b\left(A\right),\;\left(A=20\cdot \log_{10}\frac{V_{1}}{V_{0}}\right)$$

where *A* is the amplitude of the AE in decibels (dB_AE_), and *N* is the total number of AE hits. The amplitude directly reflects the damage. As a macrocrack propagates, high-amplitude signals are generated and *b* decreases (Figure 4; note the slope of the black line). The *b*-value reflects structural health, and has been used to assess signals from stochastic processes such as earthquakes and concrete structures. Furthermore, an I*b*-value that incorporates specific statistical parameters of the amplitude distribution has been derived. In I*b*-value analysis [26,27,34], the crucial factors in determining amplitude range are the mean value (*μ*), standard deviation (*σ*), lower amplitude (*μ* − a_1_∙*σ*), and upper amplitude (*μ* + a_2_∙*σ*) (Figure 4; note the slope of the blue line):

(3) $$Ib=\frac{\log_{10}N\cdot \left(\mu -a_{1}\cdot \sigma \right)-\log_{10}N\cdot \left(\mu +a_{2}\cdot \sigma \right)}{\left(a_{1}+a_{2}\right)\cdot \sigma }$$

## 3. Results and Discussion

### 3.1. Classification of Fracture Modes

Table 3 summarizes the experimental results, including the total test time, failure stress and strain, time at which the first AE signals were detected, total number of AE hits, and maximum AE amplitude. The failure stresses and strains of the CFRP specimens decreased rapidly as the fiber angle increased. The tensile stress of [0]_8_ was greater than the stress of other specimens. All specimens generated large amplitudes of AE (100 dBAE) during the last stage of tensile testing. The total number of AE hits increased in the order [90)_16_ < [80)_16_ < [0]_8_ < [0/90]_4._ In the unidirectional off-axis specimens, the fracture behavior was comparatively monotonous; the strength was also low, leading to fewer AE hits (see Table 1).

**Figure 5:**
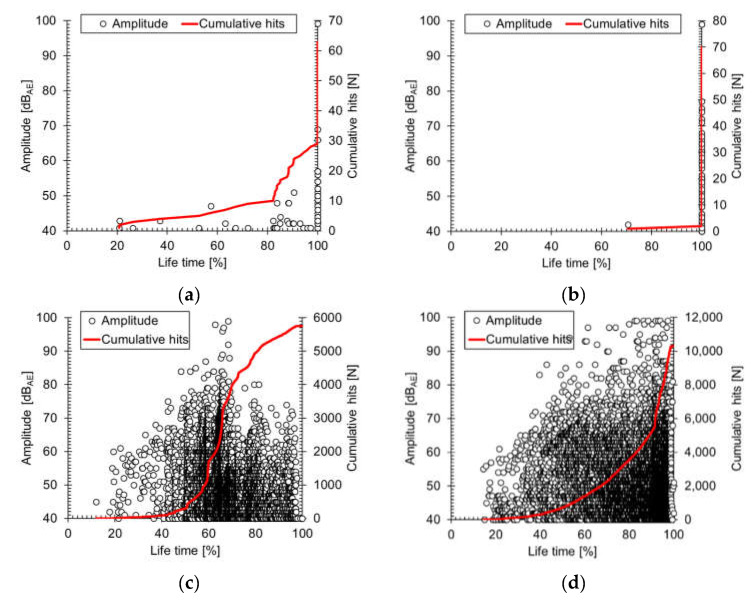
Variation in stress with change in amplitude (black dot) and cumulative hits (red line) over the lifetimes of (a): [90]_16_, (b): [80]_16_, (c): [0]_8_, and (d): [0/90]_4_ specimens under the tensile test.

Figure 5 shows the AE results for tensile testing of the [90]_16_, [80]_16_, [0]_8_, and [0/90]_4_ CFRP specimens. The horizontal axes of all illustrations represent the specimen lifetime, which was defined as 100% of the time of final failure. The AE amplitude distribution yields a unique trend corresponding to the degree of damage. Both the amplitude (black circle) and the cumulative hits (red line) changed according to the characteristics of the fractures. For example, microcracks generated a large number of hits with small amplitude from the initial stage of a tensile test. In contrast, macrocracks caused a small number of hits with large AE amplitude in the final stage of a test. In off-axis specimens (Figure 5a,b), the crack propagated immediately after matrix cracking occurred, because of weak adhesion between fiber and matrix, and the AE signal was small. However, in Figure 5c,d, all failure modes and complex failure behavior occurred. In [0]_8_, there was a maximum amplitude at 65% lifetime, and low-amplitude signals appeared after the peak. However, in [0/90]_4_, the same amplitude level was generated continuously, despite occurrence of the maximum amplitude. This phenomenon was caused by load-carrying involving the longitudinal (0-degree) layer after failure of the transverse (90-degree) layers. After failure of the transverse layers, longitudinal layers failed, under the influence of the stress concentration from existing cracks.

**Figure 6:**
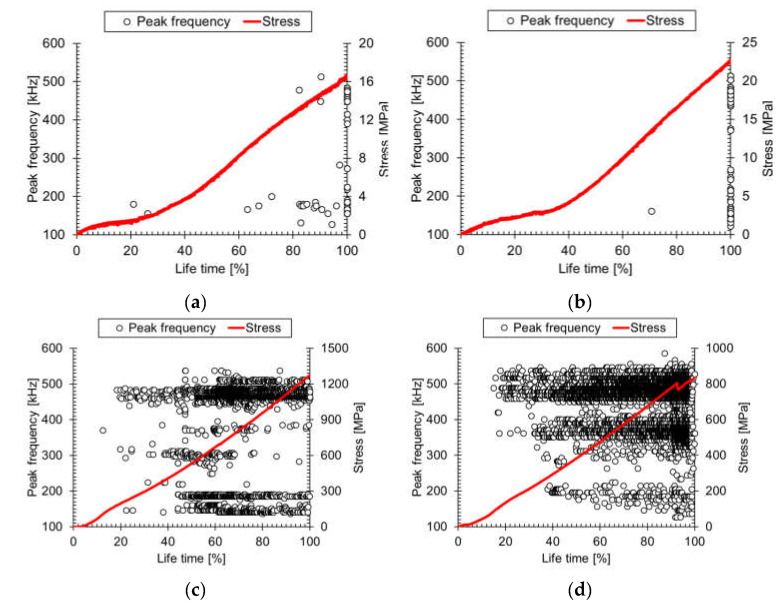
Distribution of peak frequency over the lifetimes of (a): [90]_16_, (b): [80]_16_, (c): [0]_8_, and (d): [0/90]_4_.

**Figure 7:**
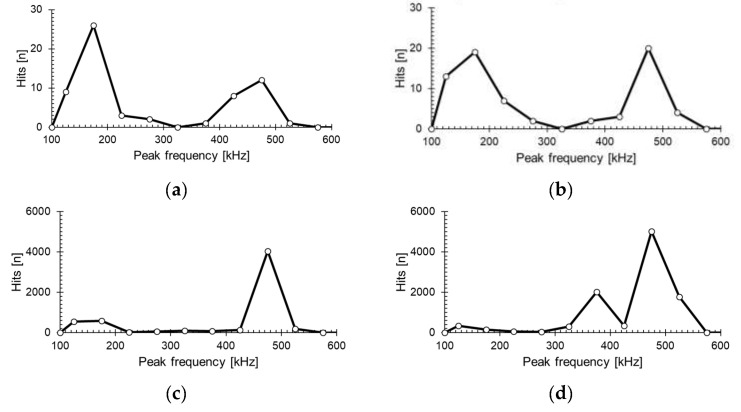
Frequency distribution of (a): [90]_16_, (b): [80]_16_, (c): [0]_8_, and (d): [0/90]_4_.

Figure 6 shows the AE hits with peak frequency. As described in Table 1, the dominant AEs in Figure 6a were caused by matrix cracking, while the dominant AEs in Figure 6b were caused by matrix cracking and fiber pull-out. As shown in Figure 6c,d, the frequency range was wide, but specific preferred frequency ranges were present. The defects tended to have distinct peak frequencies. Figure 7 shows the peak frequency as the total hits after the experiment, such that the frequency range for each damage mode is distinguishable. The frequency ranges for matrix cracking, interfacial failure, and fiber breakage were confirmed as 100–220 kHz, 300–420 kHz, and 450–600 kHz, respectively. As mentioned in the literature, matrix cracking at a low frequency is fiber fracture at a high frequency, and there is good agreement with this result. Therefore, we set the frequency according to each failure mode.

**Figure 8:**
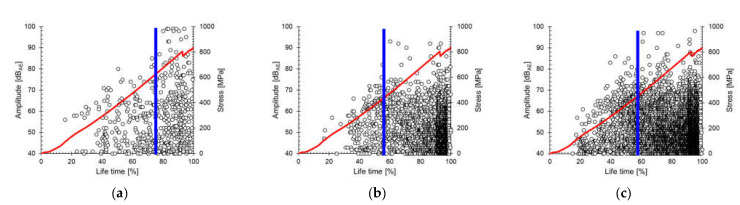
Distribution of cumulative hits according to fracture mode: (**a**) matrix cracking, (**b**) interfacial failure, and (**c**) fiber failure.

Figure 8 shows the amplitude distribution of the AEs emitted from the cross-ply specimens for each failure mode classified with the mentioned frequency ranges. The time at which the maximum amplitude occurred varied depending on the failure mode. Thus, the activation point differed among fracture modes, and previous behavior was the cause of subsequent behavior. We determined that the time points were 60% and 80%.

### 3.2. Classification of Fracture Modes

**Figure 9:**
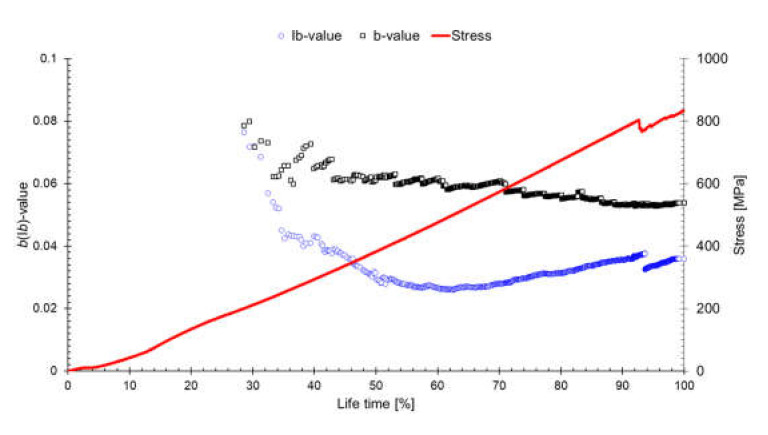
Lifetime dependency of the *b*- and I*b*-values.

Figure 9 shows the results of analyzing the *b*- and I*b*-values of the [0/90]_4_ specimen. The parametric values were calculated after generation of each set of 10 AE hits (i.e., 1st–10th, 1st–20th, …, and 1st–100th), and the slope was obtained using the least squares method.

The amplitude slope was calculated as an I*b*-value within a specific range around the mean value (a_1_ = 10; a_2_ = 5). The *b*-value decreased rapidly at the early stage, then decreased continuously (black square). The I*b*-value first decreased, but then increased after a 60% lifetime. This trend contradicts the previous literature, indicating that the complex damage behavior affected microcrack and macrocrack propagation behavior. Regardless of the fracture behavior of the material, quantitative analysis demonstrated that the initiation of low-amplitude signal (microcracking) occurred constantly. Overall, many high-amplitude signals were generated, leading to a decrease in *b*-value, but many low-amplitude signals were generated near the mean value (approximately 50 dB_AE_), and comprised the majority of signals.

**Figure 10:**
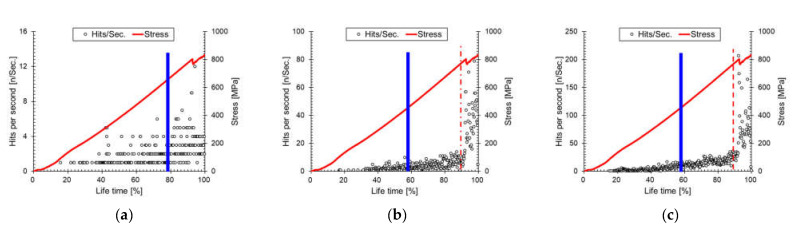
AE hits per second according to fracture mode: (**a**) matrix cracking, (**b**) interfacial failure, and (**c**) fiber failure.

The fiber structure causes significant variability in FRP structural behavior. In cross-ply composites, matrix cracking in transverse layers is the initial source of microcracks, and longitudinal layers resist axial loads. In Figure 10, AE hits per second are plotted for each damage mode. The AE hits per second increased gently in all fracture modes, then increased rapidly after a 90% lifetime (final failure). Unlike the [90]_16_ composite, the initial cracks were supported between longitudinal plies in the cross-ply composite.

This caused additional crack initiations in transverse plies at a sufficient distance from existing transverse cracks, as well as some stress concentration in longitudinal plies [35]. Concurrently, when the longitudinal fibers were splitting (compare Figure 5c with 5d), the transverse layers supported the crack. Consequently, many low-amplitude signals were generated continuously until final failure, and the high-amplitude fiber failures continued until final failure [14]. Accordingly, the I*b*-value rose again in the [0/90]_4_ composite. This fracture behavior was also confirmed in previous studies. Therefore, in the cross-ply, the hits per second increased continuously with increasing lifetime, and each fracture mode was activated continuously (see also Figure 5d).

**Figure 11:**
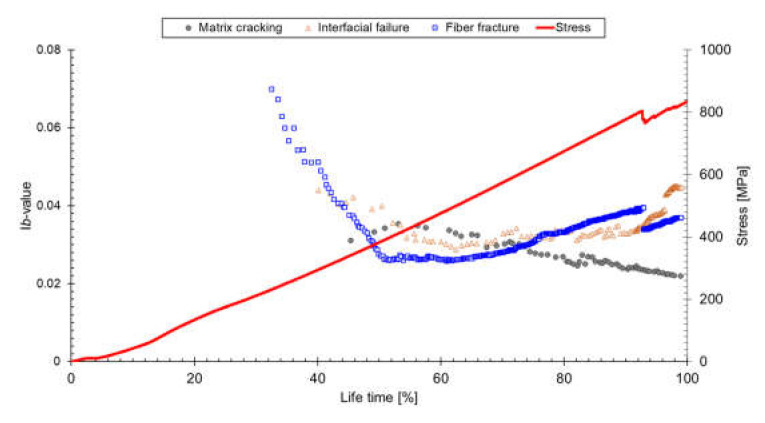
I*b*-value analysis according to fracture mode.

Figure 11 shows I*b*-values plotted according to failure mode. The I*b*-value increase trend is the microcrack-dominant phenomenon, and the I*b*-value decrease trend is the macrocrack-dominant phenomenon. In a lifetime of 55%, the I*b*-value of matrix cracking converted from positive to negative, implying that the matrix cracking began to propagate to macrocracks. The I*b*-values of fiber fracture also changed from a negative trend to a positive trend at similar stages, indicating that low-amplitude fiber fracture signals were emitted. Thus, fiber failure occurs with lower energy, and the stress concentration from existing broken fibers is the crucial factor. Interfacial failure also changed to a positive trend immediately after the fiber fracture and matrix cracking. The fiber failure and interfacial failure appeared first in composite materials, and matrix cracking linked those individual cracks, thus producing macrocracks. Consequently, the 55% lifetime is a more critical threshold in failure behavior (i.e., microcrack to macrocrack). Moreover, it is possible to detect abnormal conditions more quickly by separation according to failure mode, rather than by I*b*-value analysis using whole AEs.

## 4. Conclusions

CFRPs with various stacking sequences were tested, and *b*- and I*b*-value analyses were performed. The [90]_16_, [80]_16_, and [0]_8_ specimens were subjected to tensile tests to separate the failure modes, and the results were analyzed according to peak frequency. The results were characterized as matrix cracking (100–220 kHz), interfacial failure (300–420 kHz), and fiber fracture (450–600 kHz). The I*b*-value of the [0/90]_4_ specimen decreased to 60% of its lifetime, then increased again. This occurred because the AE hits increased without enhancing the amplitude, because of splitting during the tensile test. Analyzing the I*b*-values separated according to the failure mode, fiber fracture occurred first, followed by matrix cracking. It is possible to detect abnormal conditions more quickly by separation according to failure mode, rather than by I*b*-value analysis using whole AEs. In particular, in composite materials that exhibit complex fracture behavior—such as cross-ply materials—I*b*-value analysis for each fracture mode is more effective for behavior detection. The detailed analysis technique and quantitative evaluation technique for each fracture mode could be utilized in real-time monitoring of structural materials, such as wind turbine blades and airplane structures. However, note that this is a lab-scale study, and thus the feasibility for a larger structure would require further exploration.

## Figures and Tables

**Table 1:** Stacking sequence of fiber-reinforced plastics and dominant fracture modes [20].

Stacking Sequence	Matrix Cracking	Interfacial Failure	Fiber Fracture
[90]_16_	○	×	×
[80]_16_	○	○	×
[0]_8_	○	○	○
[0/90]_8_	○	○	○

**Table 2:** Specifications of carbon-fiber-reinforced plastic specimens (mm).

Stacking Sequence	Width	Length	Thickness	Center Hole
[90]_16_	20	200	3.6	4
[80]_16_	20	200	3.6	4
[0]_8_	16	200	1.8	6
[0/90]_8_	16	200	1.8	6

**Table 3:** Summary of experimental results.

Parameters	Unit	[90]_16_	[80]_16_	[0]_8_	[0/90]_4_
Total test time	sec	522	581	611	494
Failure stress	MPa	16.6	22.7	1275	834
Failure strain	%	0.87	0.97	1.01	0.82
First AE hit time	sec	108.72	70.61	73	71.58
Total AE hits	-	63	70	5754	10,282
Maximum. amplitude	dB_AE_	99	99	99	99

## Data Availability

Not applicable.
